# A Comparison of the Diabetes Risk Score in HIV/AIDS Patients on Highly Active Antiretroviral Therapy (HAART) and HAART-Naïve Patients at the Limbe Regional Hospital, Cameroon

**DOI:** 10.1371/journal.pone.0155560

**Published:** 2016-05-19

**Authors:** Christian Akem Dimala, Julius Atashili, Josephine C. Mbuagbaw, Akam Wilfred, Gottlieb L. Monekosso

**Affiliations:** 1 Faculty of Epidemiology and Population Health, London School of Hygiene and Tropical Medicine, London, United Kingdom; 2 Faculty of Health Sciences, University of Buea, Buea, Cameroon; Imperial College London, UNITED KINGDOM

## Abstract

**Background:**

Highly active antiretroviral therapy (HAART) has been associated with dysglycaemia. However, there is scarce data on the risk of developing diabetes mellitus (DM) in HIV/AIDS patients in Africa.

**Objectives:**

Primarily to quantify and compare the risk of having diabetes mellitus in HIV/AIDS patients on HAART and HAART-naïve patients in Limbe, Cameroon; and secondarily to determine if there is an association between HAART and increased DM risk.

**Methods:**

A cross-sectional study was conducted at the Limbe Regional Hospital HIV treatment center between April and June 2013, involving 200 HIV/AIDS patients (100 on first-line HAART regimens for at least 12 months matched by age and gender to 100 HAART-naïve patients). The Diabetes Risk Score (DRS) was calculated using a clinically validated model based on routinely recorded primary care parameters. A DRS ≥ 7% was considered as indicative of an increased risk of developing DM.

**Results:**

The median DRS was significantly higher in patients on HAART (2.30%) than in HAART-naïve patients (1.62%), p = 0.002. The prevalence of the increased DM risk (DRS ≥ 7%) was significantly higher in patients on HAART, 31% (95% CI: 22.13–41.03) than in HAART-naïve patients, 17% (95% CI: 10.23–25.82), p = 0.020. HAART was significantly associated with an increased DM risk, the odds ratio of the HAART group compared to the HAART-naïve group was 2.19 (95% CI: 1.12–4.30, p = 0.020). However, no association was found after adjusting for BMI-defined overweight, hypertension, age, sex, family history of DM and smoking (Odds ratio = 1.22, 95% CI: 0.42–3.59, p = 0.708). Higher BMI and hypertension accounted for the increased risk of DM in patients on HAART. Also, more than 82% of the participants were receiving or had ever used Zidovudine based HAART regimens.

**Conclusion:**

HIV/AIDS patients on HAART could be at a greater risk of having DM than HAART-naïve patients as a result of the effect of HAART on risk factors of DM such as BMI and blood pressure.

## Introduction

Highly active antiretroviral therapy (HAART) has greatly reduced the morbidity and mortality of HIV-infected patients [1]. However, several studies have found an association between HAART and metabolic complications such as dyslipidemia, dysglycaemia and diabetes mellitus (DM) [2–8]. Though few, some studies in Africa found no association between HAART and dysglycaemia [9,10]. In Cameroon specifically, HAART duration was not found to significantly affect glucose metabolism [11]. These findings may suggest that subjects of African descent may be less prone to developing HAART-related abnormalities of glucose metabolism.

However, it is critical to understand whether increased DM rates in patients on HAART as found in previous studies [4,6–8] are causally associated to HAART and/or its effect on the risk factors of diabetes. Many studies have assessed the association between HAART and Insulin resistance, dysglycaemia or diabetes, but few have quantified the risk of developing diabetes by assessing its traditional risk factors. A quantification of this risk of developing diabetes is therefore important because it can help identify individuals at risk, given that DM is often missed until complications set in.

In Cameroon, we found no study that assessed the risk of developing diabetes in HIV/AIDS patients. We therefore conducted this study which had as objectives to: 1. compare the median Diabetes Risk Score in patients on HAART and HAART-naïve patients at the Limbe Regional Hospital (LRH); 2. compare the prevalence of an increased risk of diabetes (DRS ≥ 7%) between patients of these two groups; 3. determine if there is an association between increased DM risk and HAART. We hypothesized that the median DRS and the prevalence of an increased risk of DM could be higher in patients on HAART than in HAART-naïve patients.

## Methods

### Study design, period and setting

This is a secondary data analysis of the hospital-based cross-sectional study that assessed the association between HAART and hypertension (HTN) in HIV/AIDS patients at the HIV treatment centre of the Limbe Regional Hospital. The Limbe Regional Hospital (LRH) is a second-level referral hospital in the South West Region of Cameroon. Participants were enrolled over a period of 3 months (8^th^ April 2013 to 21^st^ June 2013).

### Participants and sampling

The study population was made up of 200 HIV/AIDS patients receiving longitudinal care at the HIV treatment center of the LRH between April 2013 and June 2013. These participants were: A group of 100 HIV/AIDS patients who had never received HAART (HAART-naïve) randomly sampled from the HIV/AIDS patients attending the LRH during the study period; and another group of 100 HIV/AIDS patients on HAART selected by consecutive convenient sampling matched by age and gender to the HAART-naïve group. As selection criteria, we included patients aged 21 years and above who gave a written consent to take part in the study and we excluded patients with a self-reported history of DM and a self-reported use of antidiabetic medications.

### Study procedures and Variables

All participants were subjected to a face-to-face interview and a physical examination. Data was collected using a standardized questionnaire. Information on socio-demographics, smoking habit, use of anti-hypertensive medication, use of steroids, family history of DM, CD4 cell count (within past 6 months), duration of HIV infection and HAART use were obtained both from the interviews and the patients’ medical records.

The physical examination entailed measurement of height, weight, body mass index (BMI), waist and hip circumferences, waist-to-hip ratio (WHR) and the blood pressure (BP).

Primary data from the principal study assessing the association between HAART and hypertension was used to calculate the DRS. The DRS is a simple validated score, measured from characteristics and factors that are routinely recorded in primary care. As shown on Table 1, the DRS was calculated for each subject by entering the following variables into the equation of the Diabetes Risk Score model [12] at the Medscape website [13]: age, gender, use of anti-hypertensive medication, use of steroids, history of smoking, family history of DM and BMI. To minimize false negatives, we considered a DRS ≥ 7% as indicative of an increased risk of developing diabetes mellitus [14]. Griffin and colleagues found that a risk score threshold of 8% had a 50% proportion of participants above the threshold with a sensitivity of 90.9%, a specificity of 51.8% and positive and negative predictive values of 8.0% and 99.2% respectively [12].

**Table 1:** Diabetes Risk Score assessed risk factors of diabetes mellitus type 2 and formula.

Risk Factors and categories	Category values
**Age (in Years)**	-
**Gender**	
*Male*	*0*
*Female*	*-0*.*879*
**Treatment of HTN**	
*No HTN medications*	*0*
*On HTN medications*	*1*.*222*
**Use of steroids**	
*Not on steroids*	*0*
*On steroids*	*2*.*191*
**Family history of DM**	
*No first degree family members with diabetes*	*0*
*Parent or sibling with diabetes mellitus*	*0*.*728*
*Parent and sibling with diabetes mellitus*	*0*.*753*
**History of smoking**	
*Non-smoker*	*0*
*Used to smoke*	*-0*.*218*
*Smoker*	*0*.*855*
**BMI (Kg/m**^**2**^**)**	
*<25*	*0*
*25–27*.*49*	*0*.*699*
*27*.*5–29*.*99*	*1*.*97*
*≥ 30*	*2*.*518*

Diabetes Risk Score = 100 / (1 + e ^(Terms)^), where;

Terms = 6.322—Sex—Treatment of HTN—Use of Steroids–(0.063 * Age)–BMI—Smoker

### Data sources and Measurements

The use of anti-hypertensive medication and steroids, history of smoking and family history of DM were based on the participants’ self-reports and their medical records. The mean BP value of each participant was calculated as the average of two measurements taken on the right arm in a sitting position using an electronic automated and clinically validated BP monitor (Omron M2, HEM—7121—E) with a suitable sized cuff (22–34 cm), with at least 5 minute intervals of rest between measurements. HTN was diagnosed according to the WHO criteria as systolic BP ≥ 140 mmHg and/or diastolic BP ≥ 90 mmHg [15]. Weight (to the nearest 0.5 kg) was measured using a scale (BRN 9311). Participants were permitted to keep on light clothing. Height (in meters to the nearest 0.5cm) was measured using a stadiometer. Body mass index (BMI) in kg/m^2^ was calculated as weight (kg)/[height (m) X height (m)]. BMI-defined overweight was considered as a BMI between 25 to 29.9 Kg/m^2^ and BMI-defined obesity as a BMI ≥ 30 Kg/m^2^. The WHR-defined abdominal obesity was considered as WHR > 0.9 in men and WHR > 0.85 in women.

### Data management and data analysis

The data collected was entered into and analyzed using Epi Info version 7 statistical software (CDC, Atlanta, USA). For objective 1, the median diabetes risk scores in each category were compared using the Wilcoxon rank sum (Mann-Whitney) test. For objective 2, the prevalences of increased diabetes risk in each category were compared using a Chi-square test. For objective 3, logistic regression was used to assess for the association between HAART and DM risk while adjusting for risk factors. Multiple linear regression was also used to assess for the association between HAART and the log DRS. A p-value < 0.05 was considered statistically significant.

### Ethical considerations

Ethical approval was granted by the Institutional Review Board of the Faculty of Health Sciences of the University of Buea and administrative authorization by the regional delegate of public health South West region and the director of the LRH. Participants were exposed to minimal risk since all measurements done were non-invasive. Confidentiality, anonymity and privacy of all participants were guaranteed at all levels of this study.

All participants provided a written informed consent to participate in the study, which was approved by the Institutional Review Board of the Faculty of Health Sciences.

## Results

### Socio-demographic and clinical characteristics of the study population

The Socio-demographic and clinical characteristics of the 200 participants are presented on Table 2. Mean age, gender distribution, occupation and region of origin were similar in the HAART group and the HAART-naïve group, except for marital status. The mean BMI, prevalence of BMI-defined overweight, duration of HIV infection and the mean CD4 cell count were higher in the HAART group than in the HAART-naïve group, while the mean WHR and the prevalence of WHR-defined abdominal obesity were similar in both groups. All the participants of the HAART group were on first-line antiretroviral therapy including drugs of the NRTI and NNRTI classes and some participants had received more than one first-line antiretroviral regimen. The mean duration of HAART was 58.6±28.5 months. Table 3 shows the HAART regimens and the respective number of participants who ever used them since their initiation of treatment.

**Table 2:** Socio-demographic and clinical characteristics of the participants.

Characteristic	All participants(n = 200)	HAART group(n = 100)	HAART-naïvegroup (n = 100)	P-value^a^
*Age (mean ± SD*, *in years)*	39.1±9.4	40.2±8.0	38.0±10.6	0.106
*Females*, *n (%)*	140 (70%)	70 (70%)	70 (70%)	1.000
*Married*, *n (%)*	89 (44.5%)	52 (52%)	37 (37%)	**0.033**
*Unskilled occupation*, *n (%)*	26 (13%)	12 (12%)	14 (14%)	0.884
*North west region*, *n (%)*	88 (44%)	46 (46%)	42 (42%)	0.850
*BMI*^b^ *(mean ± SD*, *Kg/m*^*2*^*)*	24.1±2.9	24.8±2.8	23.5±2.8	**0.002**
*BMI-defined overweight(prevalence*, *%)*	40.5%	50%	31%	**0.006**
*WHR*^c^ *(mean ± SD)*	0.86±0.06	0.86±0.07	0.85±0.06	0.333
*WHR-defined abdominal obesity (prevalence*, *%)*	44.5%	46%	43%	0.669
*Duration of HIV infection (mean ± SD*, *in months)*	34.7±39.2	66.5±30.9	2.9±8.9	**< 0.001**
*CD4 cell count (mean ± SD*, *cells/μL)*	308±237	501±225	197±160	**< 0.001**

^a^ P-values for statistical tests comparing variables of the HAART and HAART-naïve groups

^b^BMI—Body Mass Index measured in Kilograms per meters squared (BMI-defined overweight defined as BMI > 25 Kg/m^2^).

^c^WHR—Waist-to-hip ratio. (WHR-defined abdominal obesity defined as WHR > 0.9 in men and WHR > 0.85 in women).

**Table 3:** Antiretroviral regimens used by the participants.

Antiretroviral therapy regimens	Participants who ever used the regimen
Zidovudine + Lamivudine + Nevirapine	82 (82%)
Tenofovir + Lamivudine + Nevirapine	65 (65%)
Stavudine + Lamivudine + Nevirapine	53 (53%)
Zidovudine + Lamivudine + Efavirenz	15 (15%)
Tenofovir + Lamivudine + Efavirenz	9 (9%)
Tenofovir + Emcitrabine + Nevirapine	2 (2%)
Tenofovir + Emcitrabine + Efavirenz	1 (1%)

### Diabetes Risk Score, prevalence of the increased DM risk and risk factors assessed

Table 4 summarizes the median diabetes risk score, prevalence of increased risk of diabetes and the risk factors assessed. The median Diabetes Risk Score was significantly higher in patients on HAART (2.30%, IQR: 1.13–8.28) than in HAART-naïve patients (1.62%, IQR: 0.58–4.06), p = 0.002. The prevalence of the increased DM risk (DRS ≥ 7%) was significantly higher in patients on HAART, 31% (95% CI: 22.13–41.03%) than in HAART-naïve patients, 17% (95% CI: 10.23–25.82%), p = 0.020. There was a similar history of smoking in both groups but the HAART group was found to have a higher prevalence of patients with HTN and on anti-hypertensive medications than the HAART-naïve group (p = 0.03). Also, patients of the HAART group were found to be more overweight than patients of the HAART-naïve group (p = 0.016). There was no significant difference in the reported family history of DM amongst the two groups, and no group reported use of steroids.

**Table 4:** Median and mean Diabetes Risk Score, prevalence of Increased DM risk and assessed risk factors of diabetes.

Parameter	All participants (n = 200)	HAART group (n = 100)	HAART-naïve group (n = 100)	P-value^a^
**DRS (%)**				
*Median (%)*	1.99	2.30	1.62	0.002^b^
*IQR*^c^*(%)*	0.15–6.40	1.13–8.28	0.58–4.06	
**Increased DM risk (DRS ≥ 7)**				
*Prevalence*, *n (%)*	48 (24%)	31 (31%)	17 (17%)	0.020
*95% CI*, *(%)*	18.26–30.53	22.13–41.03	10.23–25.82	
**History of smoking**				
*Smoker*	3 (1.5%)	2 (2%)	1 (1%)	0.844
*Used to smoke*	8 (4%)	4 (4%)	4 (4%)	
*Non-smoker*	189 (94.5%)	94 (94%)	95 (95%)	
**Treatment of HTN**				
*No HTN medications*	143 (71.5%)	62 (62%)	81 (81%)	0.003
*On HTN medications*	57 (28.5%)	38 (38%)	19 (19%)	
**BMI (Kg/m**^**2**^**)**				
*<25*	119 (59.5%)	50 (50%)	69 (69%)	0.016
*25–27*.*49*	50 (25%)	29 (29%)	21 (21%)	
*27*.*5–29*.*99*	31 (15.5%)	21 (21%)	10 (10%)	
**Family history of DM**				
*No first degree family members with diabetes*	189 (94.5%)	91 (91%)	98 (98%)	0.055
*Parent or sibling with diabetes mellitus*	11 (11%)	9 (9%)	2 (2%)	
**Use of steroids**^d^				
*Not on steroids*	200 (100%)	100 (100%)	100 (100%)	-
*On steroids*	0 (0%)	0 (0%)	0 (0%)	

^a^ P-value for Chi-square test comparing the HAART and HAART-naïve groups

^b^P-value for Wilcoxon rank sum (Mann-Whitney) test comparing the HAART and HAART-naïve groups

^c^IQR—Interquartile range

^d^No participant reported using steroids

### Association between the DRS, increased DM risk and HAART

In univariate analyses, being on HAART was significantly associated with an increased risk of DM, the odds ratio of the HAART group compared to the HAART-naïve group was 2.19 (95% CI: 1.12–4.30, p = 0.020) (Table 5). Using logistic multivariable regression, we adjusted for BMI-defined overweight, hypertension, age, sex, family history of DM and smoking. HAART was no more significantly associated with increased risk of DM, the adjusted odds ratio comparing the HAART group to the HAART-naïve group being 1.22 (95% CI: 0.42–3.59, p = 0.708) (Table 5). The DRS was log-transformed and a multiple linear regression model used to assess the association between the log DRS and HAART use while adjusting for BMI-defined overweight, hypertension, age, sex, family history of DM and smoking. We had a regression coefficient of 0.031 (p value = 0.710) and a correlation coefficient of 0.82.

**Table 5:** Increased DM risk odds ratios comparing HAART and HAART-naive patients.

Factor and categories	Odds ratio	95% confidence interval	P-value
*HAART / HAART-naive*	2.19	1.12–4.30	**0.020**
*HAART / HAART-naive*	1.22^a^	0.42–3.59	0.708^b^

^a^ Adjusted for age, sex, HTN, BMI-defined overweight, and family history of DM

^b^P value corresponding to the Z-statistic using the Wald test

## Discussion

In this study, we quantified and compared the risk of diabetes in HIV/AIDS patients on HAART and HAART-naïve patients by assessing the traditional risk factors of DM. We also assessed if HAART use was associated with an increased risk of having DM. We found that this risk is significantly higher in patients on HAART than in HAART-naïve patients.

Incidence rates of DM as high as 5–14 cases per 1000 person years of follow up have been reported in HIV/AIDS patients after initiation of HAART [4,16]. These alarming rates draw particular attention because multiple high-risk conditions and cardiovascular disease have been found to have worsened clinical outcomes in HIV/AIDS patients having DM as co-morbidity [5]. Hence early identification of patients at risk of DM is of utmost importance. Even though we found no study that quantified and compared the risk of having diabetes in these patients using the DRS, a Norwegian study had results similar to ours using the Framingham risk score [17]. This study found a higher Framingham risk score and mean estimated risk of cardiovascular heart disease in patients on HAART than in HAART-naïve patients, even though the Framingham risk score has been reported to be a less accurate predictor of DM than the metabolic syndrome [18].

The median DRS were significantly higher in patients on HAART than in HAART-naïve patients. Likewise, the prevalence of the increased risk of having diabetes (DRS ≥ 7) was more elevated in the patients on HAART. This means that our patients on HAART were more likely to end up having diabetes. This finding is supported by that of previous studies that reported an elevated prevalence of DM in HIV/AIDS patients receiving treatment [4,6]. Patients on HAART had a longer duration of HIV infection. It has been suggested that HIV infection could contribute to insulin resistance and consequently diabetes [5,19,20]. One of the proposed mechanisms through which HIV infection affects glycaemia is through the generalized inflammation with up-regulation of chemokines involved in insulin regulation [5]. However, duration of HIV infection was not one of the factors used in assessing the risk of diabetes in these participants. As such the difference in the duration of HIV infection between these two groups is less likely to explain the observed higher risk of DM in the patients on HAART over the HAART-naïve ones. The role of the traditional risk factors of DM in the development of DM however remains significant [5]. For the risk factors used to quantify the DRS in our study, there was no significant difference between both groups of patients in terms of the age, sex, family history of DM and smoking history. However, the patients on HAART were more hypertensive with higher body mass indices. Our findings could mean that HAART affects some risk factors such as BMI [4,21] and blood pressure [11,22–25] which predispose to having diabetes. Dave and colleagues assessed the effect of non-nucleoside reverse transcriptase inhibitors on glycaemia and found that BMI and waist circumference in HIV/AIDS patients increased as duration on HAART increased [10]. This was also observed in the Data Collection on Adverse Events of Anti-HIV Drugs (D:A:D) Study [4], a large cohort of HIV-infected patients followed-up for several years to determine the association between antiretroviral therapy and cardiovascular disease risk. BMI could increase because of the redistribution of body fat/visceral fat accumulation and body shape changes that occur after initiation of antiretroviral therapy. This increase in BMI has been associated with DM [16,20], most probably through mechanisms involving insulin resistance. Blood pressure was also found to increase as the duration on HAART increased [11], with persistently higher prevalences of HTN observed in patients on HAART compared to HAART-naïve patients [22–25]. Several mechanisms for HAART-induced elevated blood pressure values have been proposed. One of these pathways is the dyslipidemia induced by HAART that could affect blood pressure in the long run [26]. As such patients on HAART could be found to have elevated blood pressure values, which have been associated with DM. Our multivariable analyses found a significant association between HAART use and DM risk, with patients on HAART having twice the odds of having an increased risk of DM compared to their HAART-naïve counterparts. However, this association was no longer significant after adjusting for hypertension, BMI, and other risk factors of DM. Likewise HAART use was not found to be linearly associated to the log DRS after controlling for the effect of these factors. This finding indicates that being overweight and having hypertension could be the main contributors to the increased risk of diabetes in patients on HAART over HAART-naïve patients in our study. Therefore HAART can either directly increase the risk of insulin resistance through lipolysis, as earlier reported [27], or it could indirectly affect the traditional risk factors of DM. Most of the participants had used more than one HAART regimen, but Zidovudine, Lamivudine and Nevirapine combination was the regimen which most of the participants were receiving or had ever received. This finding could as well support the observed higher DM risk in the HAART group, since Zidovudine based HAART regimens have been reported to be associated with DM [4,28]. However, we were not able to determine the effect of the individual antiretroviral drugs on the DM risk since most participants had received several regimens.

However, from this study, it may not be possible to identify all the contributors to the higher risk of DM in patients on HAART compared to HAART-naïve patients. DM in HIV/AIDS patients is multifactorial in aetiology involving an interplay of the traditional risk factors of DM, HAART and other disease-related factors [5,16]. On the other hand, similar prevalences of DM in HAART and HAART-naïve patients have been reported in some studies [9,10]. In the study by Manuthu and colleagues, participants on HAART had a higher prevalence of hypercholesterolemia and high LDL cholesterol levels than HAART-naïve participants. Both groups had similar prevalences of diabetes, impaired fasting glucose and impaired glucose tolerance [9]. However, in this study, more than half of the participants in the HAART group had been on treatment for less than a year. This could partly explain why they found no difference in the prevalence of DM between these two patient groups, since HAART often requires a longer period of time to bring about pronounced metabolic changes. Likewise, Dave and colleagues reported no significant difference in the prevalence of dysglycaemia between HAART and HAART-naïve patients, but found an association between Efavirenz and dysglycaemia [10]. Nevertheless, a recent study by Araujo and colleagues revealed possibly lower insulin resistance with newer anti-retroviral regimens [20].

Our study is limited by potential bias from self-reports of the measured risk factors of diabetes by participants. The study did not control for unmeasured potential confounders such as dyslipidemia. Also, the DRS was designed based on a Caucasian study population and its applicability may be questioned in populations of a different ethnic background [29]. We therefore recommend more studies that evaluate and quantify the risk of DM in HIV/AIDS in different settings and with larger sample sizes.

In conclusion, our study found that HIV/AIDS patients on HAART are at a greater risk of having DM than age and gender-matched HAART-naïve patients. Diabetes, as a consequence of HAART or its traditional risk factors, should be diagnosed early enough in HIV/AIDS patients since it could significantly increase their mortality and morbidity.
